# Optimizing Diagnosis of Urothelial Bladder Cancer with Blue Light Cystoscopy via Recognition of False-Positive Lesions

**DOI:** 10.1089/vid.2017.0073

**Published:** 2018-04-06

**Authors:** Soroush T. Bazargani, Hooman Djaladat, Anne K. Schuckman, Cory M. Hugen, Siamak Daneshmand

**Affiliations:** Department of Urology, USC/Norris Comprehensive Cancer Center, University of Southern California, Los Angeles, California.; Department of Urology, USC/Norris Comprehensive Cancer Center, University of Southern California, Los Angeles, California.; Department of Urology, USC/Norris Comprehensive Cancer Center, University of Southern California, Los Angeles, California.; Department of Urology, USC/Norris Comprehensive Cancer Center, University of Southern California, Los Angeles, California.; Department of Urology, USC/Norris Comprehensive Cancer Center, University of Southern California, Los Angeles, California.

**Keywords:** blue light cystoscopy, urothelial bladder cancer, false-positive, transurethral resection of bladder cancer

## Abstract

***Introduction:*** Blue light cystoscopy (BLC) using hexaminolevulinate (Cysview^®^) improves the detection of nonmuscle invasive bladder cancer (NMIBC).^1–3^ BLC results in lower recurrence rate and a better recurrence-free survival, as well as a progression benefit.^4^ However, false-positive (FP) fluorescence can occur for various reasons and can vary among different series. Studies have shown that FP rates are not significantly different from white light (WL) cystoscopy. We evaluated different scenarios producing FP in BLC.

***Methods:*** Under institutional review board approval, we prospectively enrolled consecutive patients undergoing transurethral resection of bladder lesions into a BLC registry between April 2014 and December 2016. Several cases are highlighted in the video demonstrating cystoscopic view under WL and blue light in specific circumstances increasing the chance of detecting an FP lesion.

***Results:*** BLC with Cysview is demonstrated in several challenging cases for the detection of NMIBC. Possible FP scenarios include tangential views of the bladder neck or side walls (1) trigone, trabeculations, or diverticula; (2) in setting of inflammation like cystitis; (3) postintravesical therapy, that is, <6 weeks interval from prior bacillus Calmette-Guérin (BCG); (4) prior resection within 6 weeks; (5) bright tiny spots; and (6) site of ureterectomy/bladder cuff resection, early fading lesions (after irrigation). Unnecessary biopsy of these lesions can be avoided through simple techniques such as changing the angle of the cystoscopic view, several rounds of irrigation, and avoiding BLC too early after BCG instillation or prior resection.

***Conclusions:*** Use of BLC with Cysview can help with the detection of NMIBC as well as carcinoma *in situ* in patients undergoing transurethral resection of bladder tumor for bladder cancer. The reported FP rates of BLC will decrease with experience and recognition of the mentioned scenarios.

Prior presentation: None.

No competing financial interests exist.

Runtime of video: 7 mins 16 secs

## Graphical abstract

**Figure:**
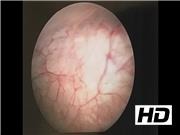

**Figure:**
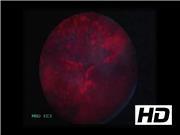

**Figure:**
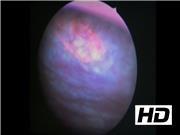

## Video

## Files

**PDF:** 

**HD:** 
